# Intracellular Replication Inhibitory Effects of Tea Tree Oil on Vesicular Stomatitis Virus and Anti-inflammatory Activities in Vero Cells

**DOI:** 10.3389/fvets.2021.759812

**Published:** 2021-11-17

**Authors:** Qi Shao, Junjie Huang, Jingui Li

**Affiliations:** ^1^College of Veterinary Medicine, Yangzhou University, Yangzhou, China; ^2^Jiangsu Co-innovation Center for Prevention and Control of Important Animal Infectious Diseases and Zoonoses, Yangzhou, China; ^3^Joint International Research Laboratory of Agriculture and Agri-Product Safety, The Ministry of Education of China, Yangzhou University, Yangzhou, China

**Keywords:** tea tree oil, vesicular stomatitis virus, inflammatory cytokines, antivirus effect, viral replication

## Abstract

Viral disease management has been proven difficult, and there are no broadly licensed vaccines or therapeutics. Vesicular stomatitis virus (VSV) is an active pathogen of wild ungulates and livestock; its infection frequently caused irreversible vesicles on the tongue or other positions, leading to enormous economic loss. Tea tree oil (TTO) has been shown to be a popular remedy for many skin diseases owing to its antibacterial, antipruritic, and anti-inflammatory effects. However, the potential effect of TTO on VSV proliferation and the corresponding inflammatory response in cells remain unclear. In this study, methyl thiazolyl tetrazolium assay was used to evaluate the cell viability of TTO, and cytotoxic concentration 50 (CC50) was calculated. Then, fluorescence observation, reverse transcription–quantitative polymerase chain reaction, Western blot (WB), and flow cytometry (FCM) assay were used to evaluate the antiviral effect of TTO against VSV under three manners of pre-infection before medication, co-administration, pretreatment before infection at safe doses to Vero cells. Meanwhile, the mRNA expressions of interleukin 8, tumor necrosis factor α, and ISG56 in cells were also detected. The results showed that the maximum safe concentration of TTO to Vero cells was 0.063% and the CC50 is 0.32%. Most notably, TTO dose-dependently inhibited the VSV GFP fluorescence generation and restrained the replication of VSV in gene and protein levels regardless of the treatment modes. Based on the results of the FCM, effective concentration 50 of TTO against VSV is 0.019%. Similarly, the mRNA expression of the above cytokines induced by viral infection was also remarkably curbed. These findings suggest that TTO emerged blocking, prophylaxis, and treatment action against VSV replication and suppressed the related inflammation in Vero cells. This study provides a novel potential for TTO fighting against viral infection and anti-inflammatory activities in Vero cells.

## Highlights

- The maximum safety concentration of Tea tree oil (TTO) to Vero cells is 0.063%.
- TTO exerted significantly inhibitory effect on the replication of VSV in Vero cells.
- Anti-VSV effect of TTO included disinfection, prophylaxis and treatment effect.
- TTO can alleviate the inflammation induced by virus infection, even recover to normal level.

## Introduction

Vesicular stomatitis (VS) is an infrequent yet important vesicular disease of livestock, caused by vesicular stomatitis virus (VSV), which is a negative strand RNA and enveloped virus. It is highly contagious and zoonotic, frequently leading to vesicles and unclear on the tongue, papilla, or other positions. Although its morbidity is not so high, the injury is irreversible, then resulting in the enormous economic losses (1, 2). VS is classified as a list B disease by Office International Des Epizooties (OIE), belonging to 1 of the 13 foreign diseases that need to be supervised in China. Because of its economic and public health importance, attempting to dig out some novel antiviral compounds to eradicate the VSV is urgent.

Natural medical plants have been applied to defend infectious diseases including viral infection with a long history and gradually considered as the storehouse for the discovery of potential antivirus agents. Among various kinds of bioactive components researched, volatile oils, also known as essential oils, are revealed to have enormous potential of pharmaceutical value, including but not limited to, antimicrobial, anti-inflammation, antioxidant, and antiparasitic activities (3, 4). For the past few years, researchers verified some essential oils such as garlic oil, which have activities against various kinds of viruses covering herpes simplex virus (HSV), HIV, and many other viruses, primarily working through inhibiting the entrance of virus (5). Similarly, tea tree oil (TTO) is verified as a multifunctional natural essential oil mainly extracted from Australia tea tree (*Melaleuca alternifolia*), composed of terpinen-4-ol, terpinene (γ-type, α-type), and approximately 100 other components (6). TTO is traditionally used as an antiseptic medicine and promoted for external use. Similar to some other known essential oils, TTO also shows the excellent antibacterial, antifungal, antiprotozoan, and anti-inflammatory effect (7–9). However, the antiviral effect of TTO remains not very clear yet.

Because of the rapid replication speed and high output of VSV, it is hard to be eradicated (10). Therefore, the extent of the viral replication is the direct index of the antiviral judgment. Besides, virus-induced secretion of cytokines, such as tumor necrosis factor α (TNF-α), types I and II interferon (IFN), and interleukin (IL)-4/8/10, are evidenced to participate in the process of virus infection (11, 12). TNF-α is a proinflammatory cytokine involved in cell immune (13). Cell process impacted by TNF-α incorporates apoptosis, cellular proliferation, inflammation, and immune regulation. High level expression of TNF-α in plasma and tissues involved in inflammation induced by HIV infection was found (14). Interferon stimulated gene 56 (ISG56) is one protein with tetratricopeptide repeats genes and always highly upregulated by viral infection or type I IFNs (15). Overexpression of ISG56 reversed cytoplasmic poly(I:C) induced inhibition of VSV replication, whereas knockdown of ISG56 inhibited VSV replication (16). IL-8 is a neutrophil chemoattractant and plays a causative role in recruiting and activating neutrophils, leading to acute inflammation (17). IL-8 has a large induction amplitude during virus infections and may be the most remarkable triggered cytokine in SARS-CoV-2 infection (18).

To explore the potential antiviral activity and features, we paid attention to the inhibitory effect of TTO on VSV replication and the corresponding inflammatory responses in Vero cells under three different treatment manners.

## Materials and Methods

### Chemical Reagents, Cells, and Virus

TTO was purchased from Jiangxi Zhonghuan New Materials Co. Ltd. (Jiangxi, China). 0.25% EDTA–tyrisin (with phenol red) was purchased from Solarbio Life Sciences (Beijing, China). Anti-GFP mouse monoclonal antibody (mAb) and anti–β-actin rabbit mAb was both purchased from TransGen Biotech (Beijing, China). Horseradish peroxidase (HRP)–conjugated goat anti–mouse immunoglobulin G (IgG) and HRP-conjugated goat anti-rabbit IgG were both purchased from Sangon Biotech (Shanghai, China). Vero cells were maintained in Dulbecco modified eagle medium (DMEM) (Hyclone Laboratories, Logan, UT, USA) containing 10% fetal bovine serum (FBS, Grand Island, NY, USA) and 100 IU/mL penicillin plus 100 g/mL streptomycin. VSV-GFP was proliferated in Vero cells and stored in −80°C.

### Cell Viability Assay

Effect of TTO to Vero viability was detected in triplicate with methyl thiazolyl tetrazolium (MTT) assay (Beyotime, China). Vero cells were seeded in a 96-well culture plate and incubated at 37°C under 5% CO_2_ with complete DMEM medium. When cell confluence reached 80%, several concentrations of TTO were applied, respectively, for further 24-h incubation under the same conditions. Ten microliters of MTT (5 mg/mL) per well was added into the plate to terminate the proliferation and incubated for 4 h. Then 100 μL dimethyl sulfoxide was added to dissolve the purple crystals after centrifugation. Optical density was then performed by microplate reader (Epoch, Biotek, USA) at 570 nm. The CC50 value was taken to be the TTO concentration at which cell viability was reduced by 50%. GraphPad Prism was applied to calculate the CC50.

### Influence of TTO on VSV Replication

**Figure 1:**
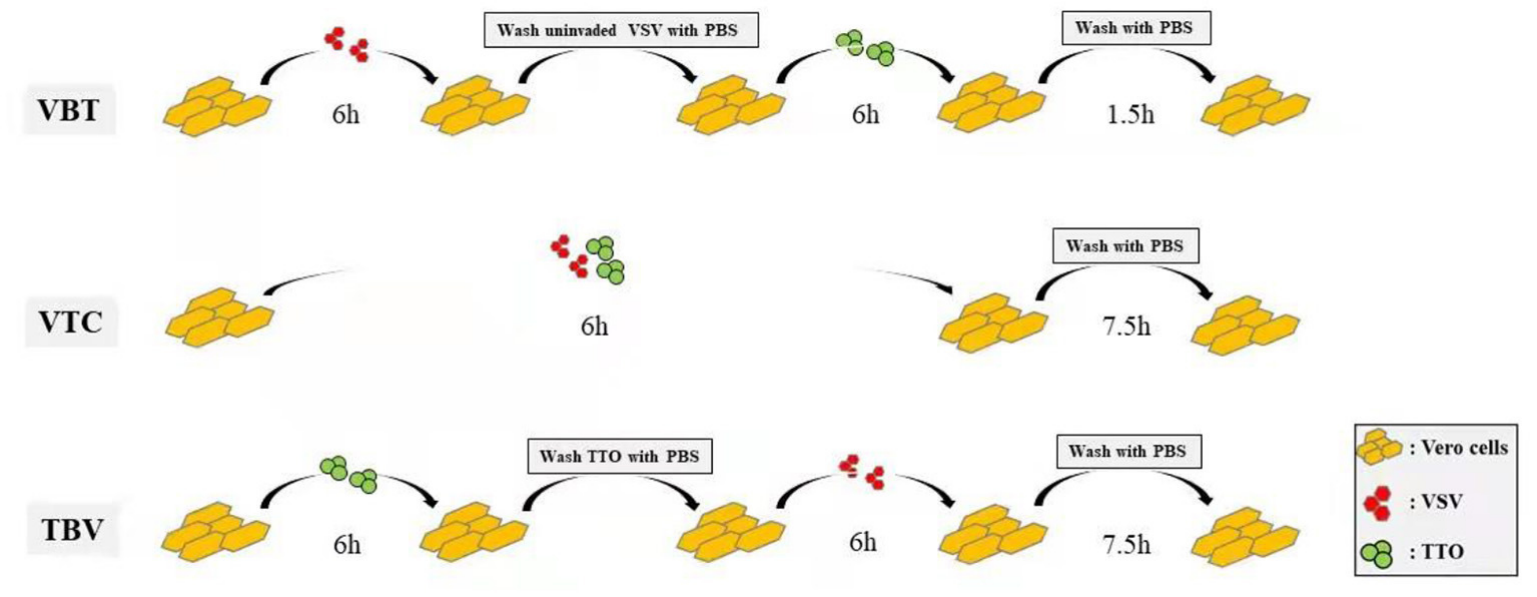
Diagram showing the different ways of experimental protocol.

Before virus or remedy administration, 5 × 10^5^ cells/well resuspended in 10% FBS DMEM were seeded in six-well culture plates and incubated at 37°C under 5% CO_2_ for 24 h. Then antiviral effect of the TTO on VSV was evaluated with different TTO concentrations [0.063%, 0.031%, 0.016%, 0.008%, 0.004% (wt/wt)] under three different treatment manners (the concentration had no toxicity to Vero cells as determined by a pre-experiment cell viability test). Preinfection with virus before TTO medication (VBT): 100 TCID_50_/well virus was challenged to Vero cells and inoculated for 6 h, and then TTO was applied to cells for another 6-h incubation after the left uninvaded virus was washed by phosphate-buffered saline (PBS) for three times gently. Virus and TTO co-administration (VTC): A mixture of 100 TCID_50_/well virus and TTO were added into the Vero cells at the same time for 6-h incubation. Premedication with TTO before virus infection (TBV): TTO was applied on Vero cells for 6-h incubation first, and then 100 TCID_50_/well virus was challenging after the TTO was washed with PBS for three times gently. The three manners, respectively, stand for the research of therapeutic effect, blocking effect, and prophylactic effect of TTO. All experiments were terminated at 13.5 h after inection and the protocol was presented as the diagram in Figure 1. Both the TTO and viruses were solved in maintenance medium (DMEM plus 2% FBS); the fluorescence generation statuses were recorded by the fluorescence microscope (IX53, Olympus, Japan). Each experiment was performed in triplicate.

### Antivirus Effect of TTO *in vitro*

The effect of TTO against VSV in Vero cells was assessed in four aspects, namely, microscopy and flow cytometry (FCM) assay for fluorescence observation and detection, reverse transcription–quantitative polymerase chain reaction (RT-qPCR) for VSV glycoprotein mRNA expression and WB for GFP protein translation.

#### Influence of TTO on VSV GFP Fluorescence Expression

FCM assay was applied to detect the relative fluorescence intensity, which stands for the replication degree of the VSV-GFP. Before FCM analysis, the trypsinized Vero cells were washed with PBS twice and then resuspended in 1 mL PBS and filtered with a 200-mesh nylon strainer. The results of FCM from group TBV was applied to calculate the effective concentration 50 (EC50) with GraphPad Prism after relative inhibitory ratio was obtained.

#### Influence of TTO on VSV RNA Synthesis

Total RNA was extracted with TRIzol reagent (R701-02, Vazyme, Nanjing, China) according to the manufacturers' instruction. After measuring the concentration and purity with NanoDrop spectrophotometer (Thermo Scientific, Wilmington, DE, USA), first-strand cDNA was synthesized according to the HiScript Q RT SuperMix kit (R123-01, Vazyme, Nanjing, China). Real-time qPCR was performed with a CFX connect real-time PCR system (Bio-Rad, USA) with 2 × ChamQ SYBR Green qPCR Master Mix (Q311-02, Vazyme, Nanjing, China). In addition, the primers for target genes are listed in Table 1. β-Actin was selected as an internal control. Primer sequences were synthesized by Tsingke Biotechnology Co., Ltd. 2^−ΔΔ*Ct*^ was applied to calculate the relative expression of mRNA, ΔCt = CT values of target gene – CT values of housekeeping gene, ΔΔCt = ΔCt of the treatment groups – *sΔ*Ct of the control group.

**Table 1:** Primers listed for VSV glycoprotein mRNA detection.

**Gene**		**Sequence 5** ^**′**^ **-3** ^**′**^
β-Actin	F	AGAAGATGACCCAGATCATGTTTGA
	R	TCCATCACGATGCCAGTGGTA
VSV	F	GAGGAGTCACCTGGACAATCACT
	R	TGCAAGGAAAGCATTGAACAA

#### Influence of TTO on VSV GFP Protein Expression

Total proteins were extracted from cells with precooled RIPA lysis buffer (C1053, APPLYGEN, Beijing, China), and the concentration of the whole proteins was quantified with Enhanced BCA Protein Assay Kit (P0010, Beyotime Institute of biotechnology, Shanghai, China). After boiling with 4 × loading buffer for 5 min, equal amounts of denatured protein in each sample (20 μg) were prepared for electrophoresis on 12% sodium dodecyl sulfate–polyacrylamide gel electrophoresis and then transferred onto polyvinylidene fluoride membranes. The immunoblot was blocked with 5% skim milk for 1.5 h at room temperature, followed by overnight incubation with primary antibodies (both diluted 1:1,000) at 4°C. After washing with Tris-buffered saline 0.1 Tween 20 (TBST), the membranes were incubated with species-specific HRP conjugated secondary antibodies (diluted 1:10,000) at room temperature for 2 h. The HRP signals were detected with enhanced chemiluminescence (ECL, E412-02, Vazyme, Nanjing, China) substrate after washing with TBST and quantified by ImageJ after developing. Moreover, β-actin was applied as the reference protein. Each experiment was performed in triplicate.

### Influence of TTO on IL-8, ISG56, and TNF-α mRNA Expression

RT-qPCR was applied to detect the virus infection–induced IL-8, ISG56, and TNF-α mRNA expression; experiment protocol is the same as that described in *Influence of TTO on VSV RNA Synthesis*. The primers for target genes were listed in Table 2. β-Actin was selected as an internal control.

**Table 2:** Primers listed for RT-qPCR in this study.

**Gene**		**Sequence 5** ^**′**^ **-3** ^**′**^
β-Actin	F	AGAAGATGACCCAGATCATGTTTGA
	R	TCCATCACGATGCCAGTGGTA
IL-8	F	TTCCTGCTTTCTGCAGCTCTGT
	R	TGGTCCACTCTCAATCACTCTCAGT
ISG56	F	GCATCACCTTCCTCTGGCTAC
	R	GCCATCTCAAATGTGGGCCT
TNF-α	F	CATGTTGTAGCAAACCCTCAAGC
	R	ATGGCACCACCAGCTGGTTAT

### Statistical Analysis

Data are expressed as mean ± standard deviation. Statistical analyses were performed by one-way analysis of variance test in GraphPad Prism 8.2.1. *p* < 0.05 stands for significant difference.

## Results

### Cell Viability and CC50 of TTO

**Figure 2:**
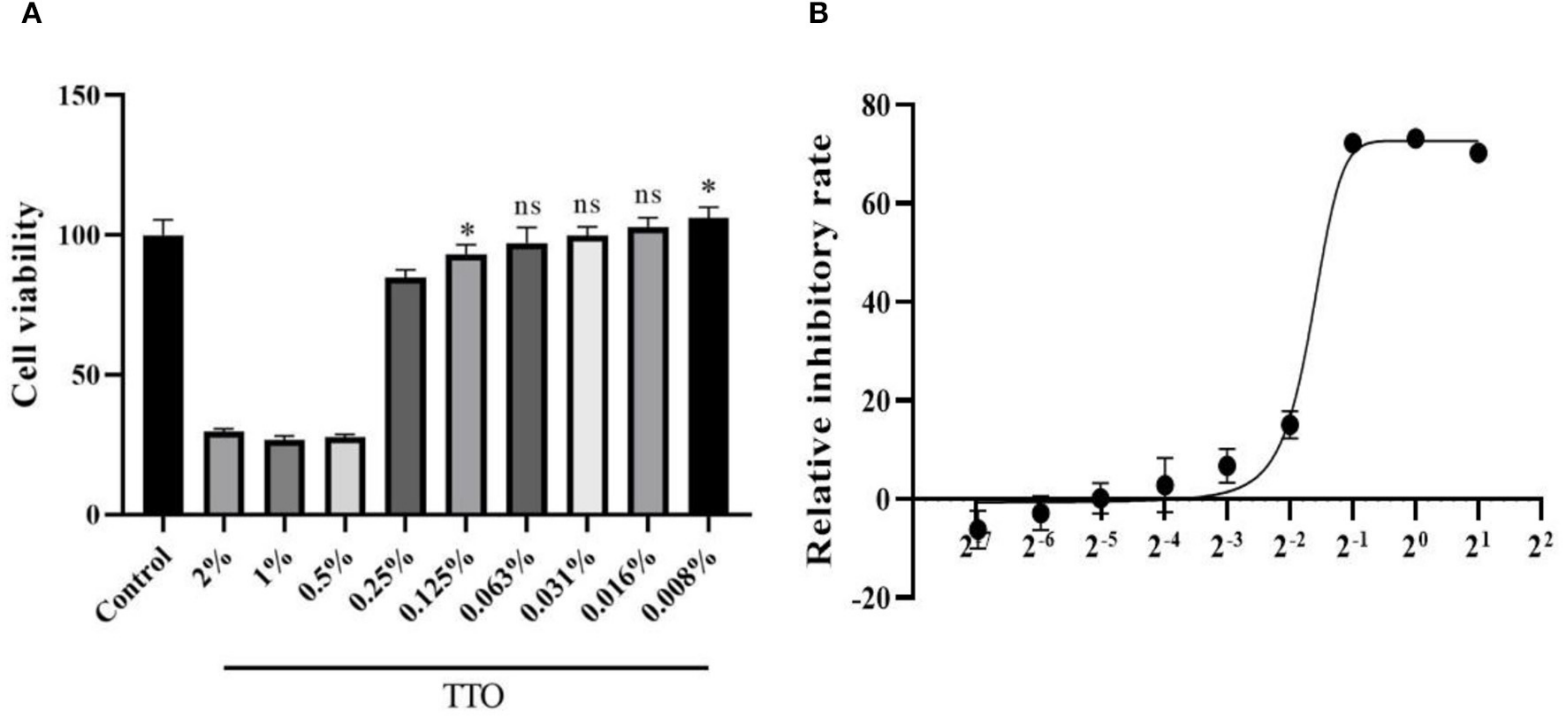
Effects of different dose of TTO to Vero viability **(A)** and dose response curve of TTO **(B)**. *Means remarkable difference *p* < 0.05; ns, no significant difference (compared to the control group).

MTT assay showed that TTO could significantly inhibit cell proliferation and activity at doses higher than 0.063% (vol/vol), and the doses no more than 0.063% can be considered as safe concentrations. Therefore, 0.063% TTO was considered as the maximum safe concentration to Vero cells. In addition, 0.008% TTO showed an obvious proliferative effect compared to control group. Cytotoxic concentration 50 (CC50) of TTO is 0.32% (Figure 2).

### TTO Inhibited the VSV GFP Fluorescence Expression in Vero Cells

**Figure 3:**
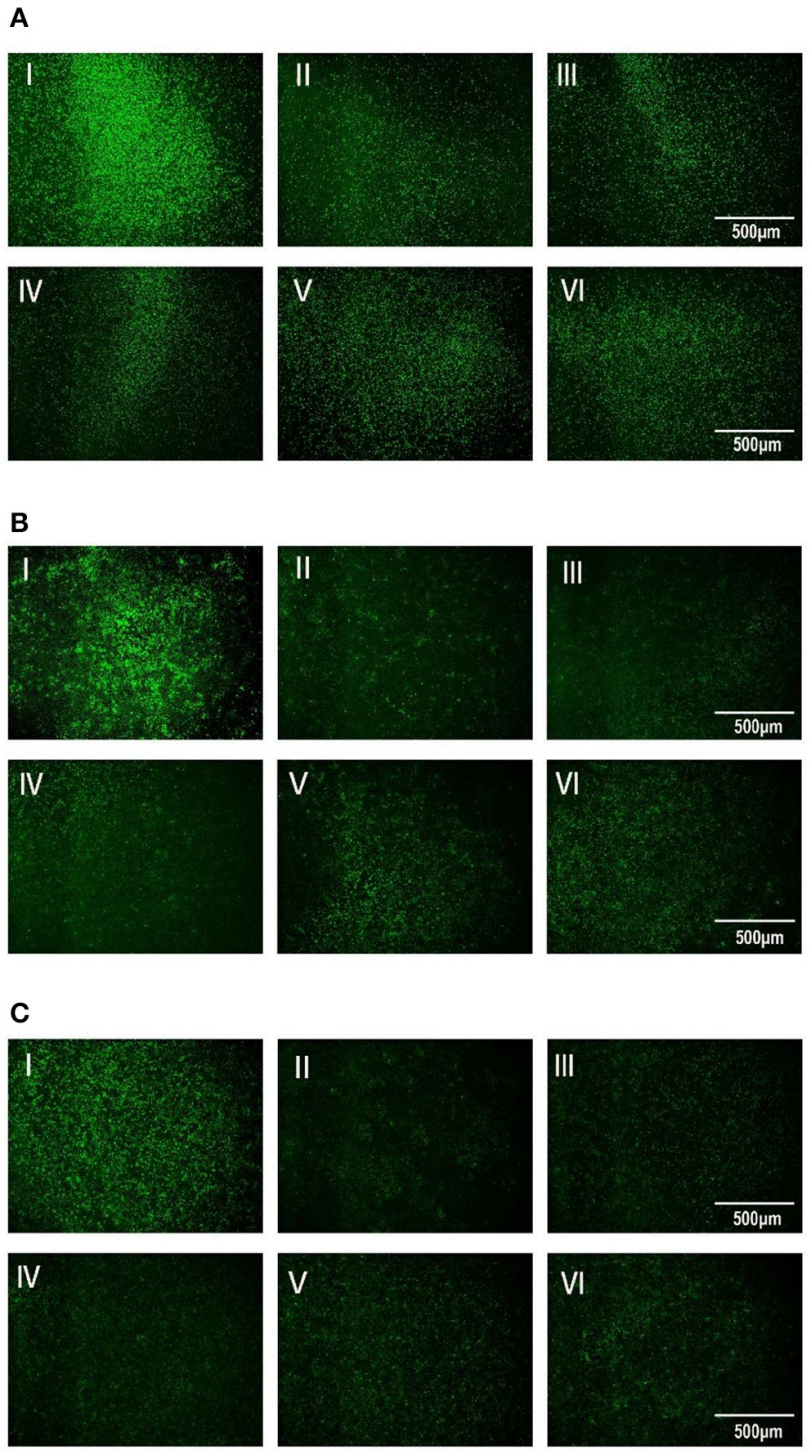
**(A–C)** The inhibitory effects of TTO on VSV GFP fluorescence expression was observed under 40 × fluorescence microscope. A: VBT; B: VTC; C: TBV; I: infected control group; II: 0.063%; III: 0.031%; IV: 0.016%; V: 0.008%; VI: 0.004%.

After 13.5-h cultivation, the amount of Vero cells in each well had no remarkable difference. Under VBT treatment as shown in Figure 3A, VSV GFP fluorescence was the strongest in Vero cells without TTO, indicating the massive proliferation of virus in cells. However, all the TTO treatments dramatically inhibited the virus proliferation, and this inhibitory effect was in a profound dose-dependent manner, suggesting the therapeutic action of TTO against VSV. For the VTC, TTO showed the similar roles on copies of VSV in Vero cells as shown in Figure 3B, indicating the blocking action of TTO on virus. Similar inhibitory effect of TTO on VSV in TBV is shown in Figure 3C, revealing the favorable prophylactic impact.

**Figure 4:**
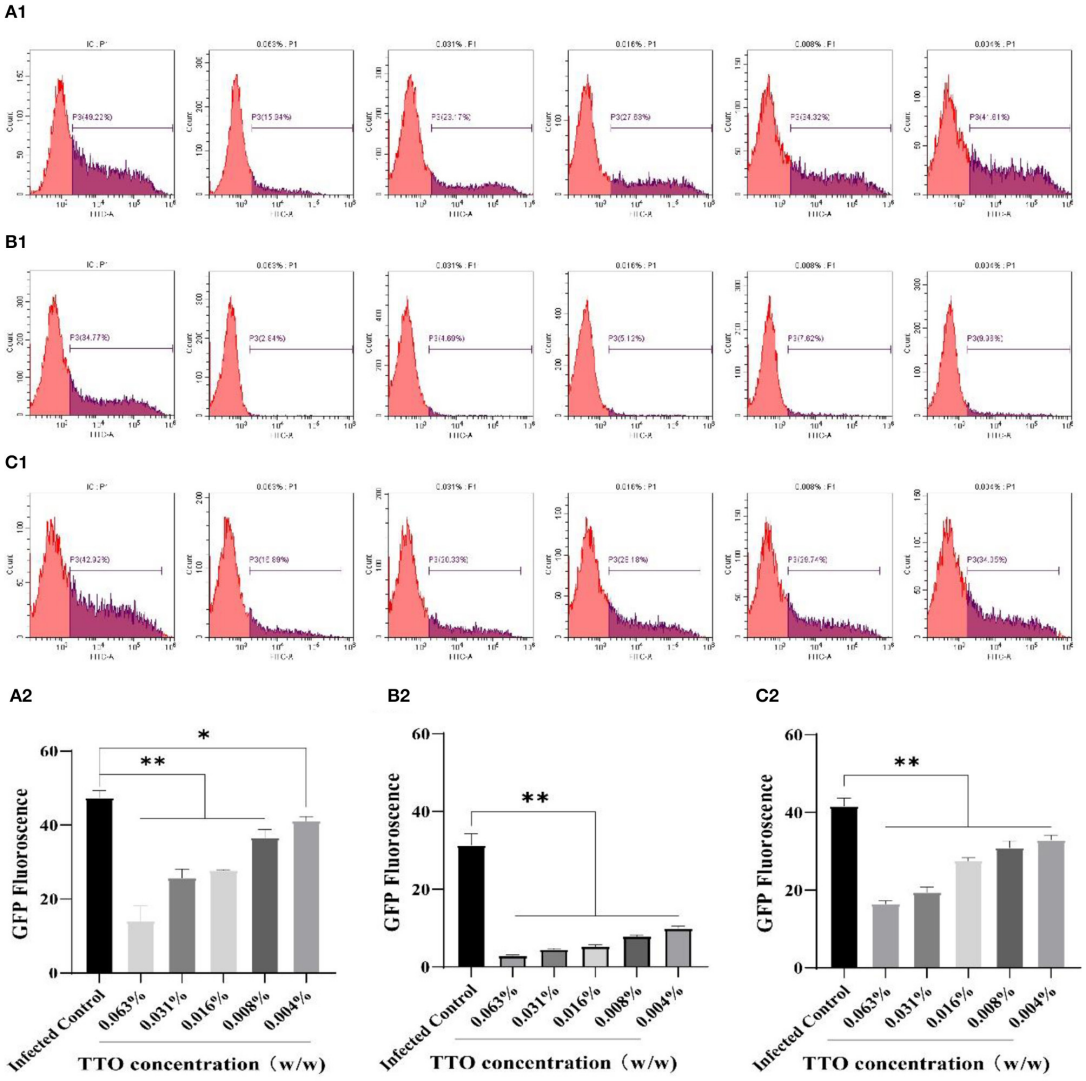
The inhibitory effects of TTO on VSV GFP fluorescence expression. Vero cells were collected and analyzed by FCM. A: VBT; B: VTC; C: TBV; **(A1,B1,C1)** P3 represented the infected cells. **(A2,B2,C2)** the rate of the GFP fluorescence in each group. **Highly remarkable difference, *p* < 0.001; *remarkable difference, *p* < 0.05.

As shown in Figure 4, the FCM analysis showed that TTO had notably dose-dependent effects on the VSV replication in three different treatment modes, consistent with the fluorescence microscopy results. Compared with infected control group, 0.063% TTO manifested a 33.28% treatment effect, 31.93% blocking effect, and 26.03% prophylaxis effect, respectively. However, the lowest dose (0.004%) TTO just had 7.61% treatment effect, 24.81% blocking effect, and 8.77% prophylaxis effect (Figures 4A1,B1,C1). The fluorescence rate of infected control group in VTC group was only 34.77%, really lower than that in the other two manners, which may be caused by the volatility of TTO. In general, blocking effect was the best anti-VSV effect of TTO.

### TTO Inhibited the Replication of VSV in Vero Cells

**Figure 5:**
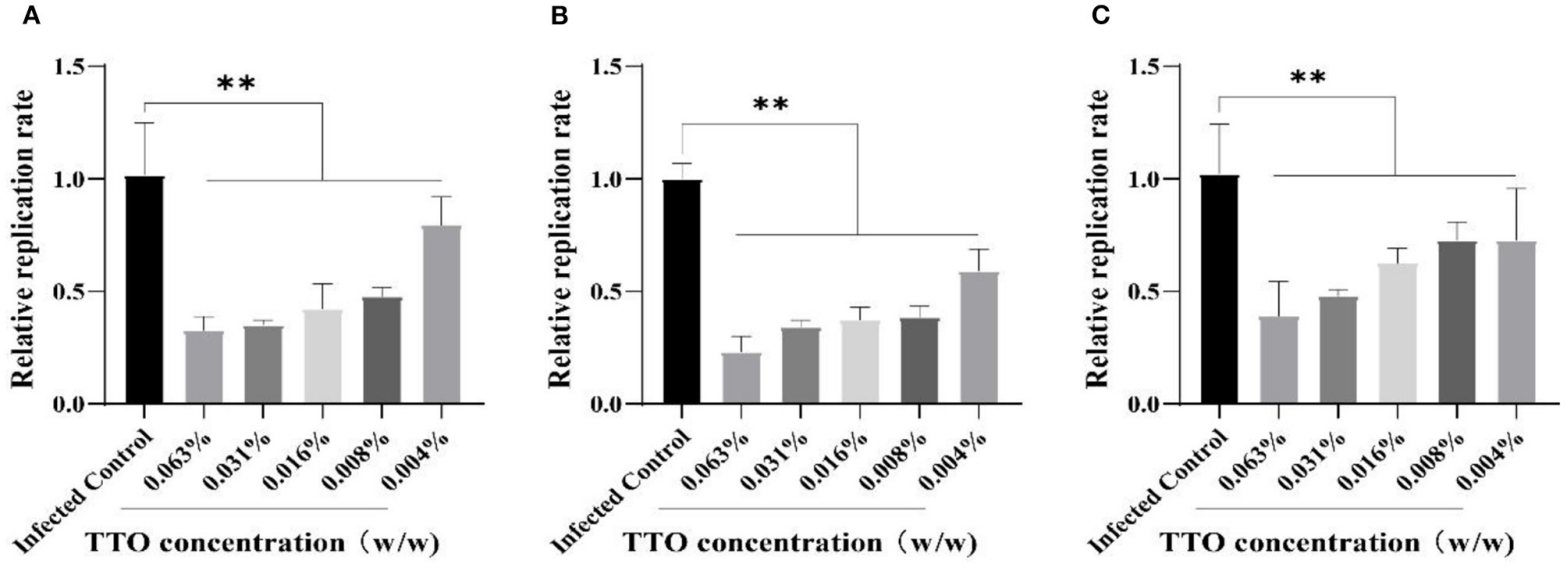
The inhibitory effects of TTO on the replication rate of VSV genome. **(A–C)** The mRNA were detected by RT-qPCR. A: VBT; B: VTC; C: TBV. **Highly remarkable difference, *p* < 0.001.

To confirm the inhibitory effect of TTO on VSV replication in Vero cells, RT-qPCR was applied to detect the relative expression of VSV genome. The result showed that TTO treatment at different doses remarkably restrained the proliferation of VSV, and the effect also exhibited an obvious dose-dependent character regardless of treatment modes as shown in Figures 5A–C. Relatively, the VTC treatment mode obtained the best suppression effect.

### TTO Inhibited the GFP Protein Expression in Vero Cells

**Figure 6:**
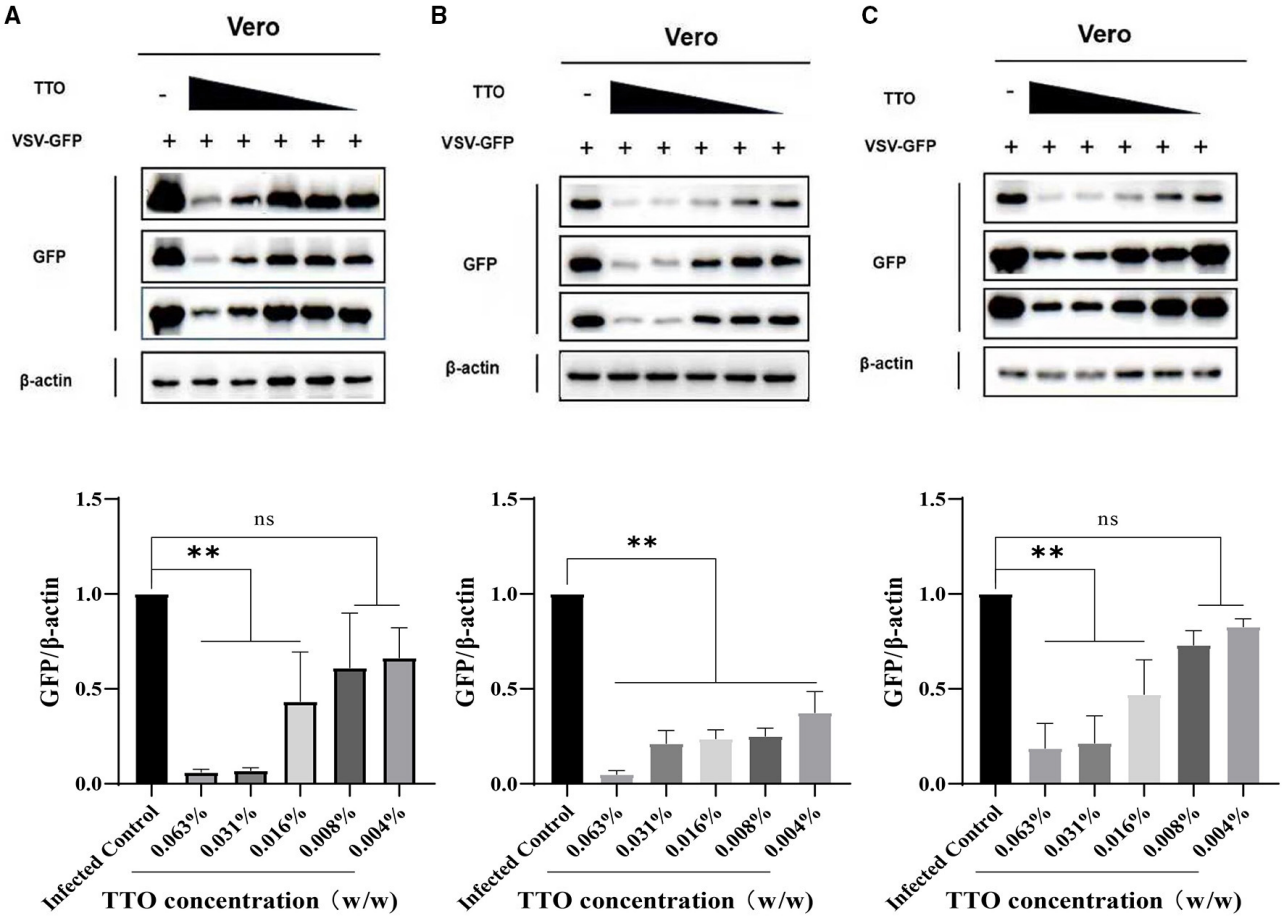
**(A–C)** VSV-GFP protein expression was inhibited by TTO. A: VBT; B: VTC; C: TBV. **Highly remarkable difference, *p* < 0.001, ns, no significant difference.

For further confirmation of the inhibitory effect of TTO on VSV GFP protein, WB analysis was performed; β-actin was chosen as the housekeeping protein. As shown in Figure 6B, the expression of VSV-GFP was strictly down-regulated by five different concentrations of TTO treatment with a dose-dependent manner compared to the infected control group, whereas in the other two manners, preinfected with virus and pretreated with TTO, both showed less effective than VTC (Figures 6A,C). Lower concentrations (0.008%, 0.004%) had no significant effect on the replication of virus.

A: VBT; B: VTC; C: TBV.

### TTO Inhibited Viral Infection–Induced Cytokines mRNA Expression

**Figure 7:**
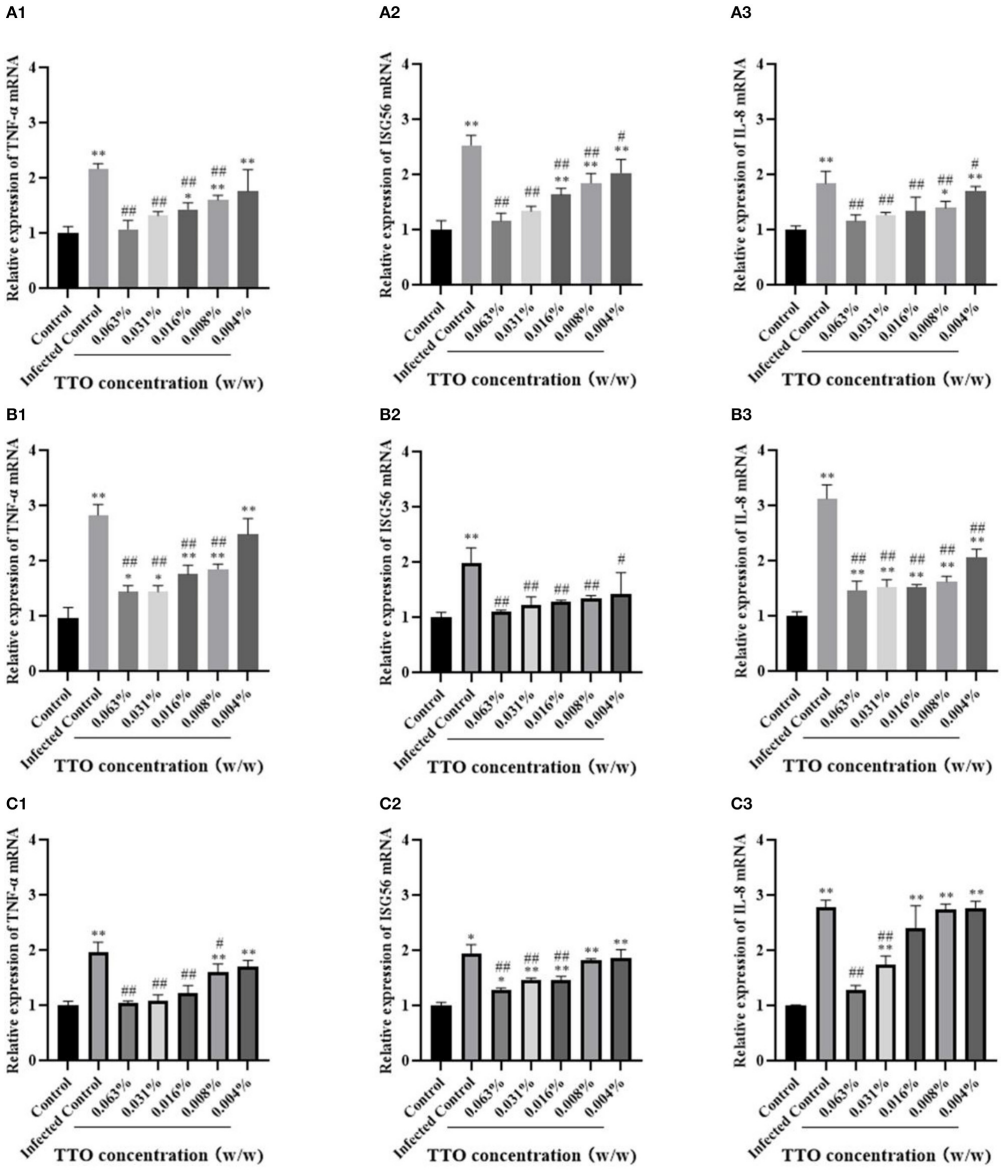
Anti-inflammatory activities were involved in the process of anti-VSV of TTO. Relative expression rates of TNF-α, ISG56, and IL-8 mRNA in Vero cells were detected by RT-qPCR. **A**: VBT; **B**: VTC; **C**: TBV. 1: TNF-α 2: ISG56; 3: IL-8. ^*##*^Highly remarkable difference, *p* < 0.001; ^#^Highly remarkable difference, *p* < 0.05 (compared to the infected control group); **highly remarkable difference, *p* < 0.001; *highly remarkable difference, *p* < 0.05 (compared to the control group).

To detect the TNF-α, ISG56, and IL-8 mRNA expression in different groups, the RT-qPCR assay was performed, and no infect-and-treat control group or infected control group were, respectively, set. Briefly, whatever the mode of treatment, TTO treatment was capable of inhibiting the rising trend induced by viral infection, exhibiting a dose-dependent character (Figure 7). As expected, the relative expression of these three viral infection–induced secretion of cytokines TNF-α (except 0.004%), ISG56 and IL-8 were dramatically down-regulated by TTO treatment in VSV preinfection modes. Compared to the control group, the relative expression of TNF-α (0.063% & 0.031%), ISG56 (0.063% & 0.031%) and IL-8 (0.063%, 0.031% & 0.016%) have no significant difference (Figure 7A). TTO and VSV co-incubation showed notable down-regulation of relative mRNA expression of TNF-α (besides 0.004%), ISG56 and IL-8, with comparison to infected control group (Figure 7B). Compared to the control group, the expression of ISG56 mRNA had no significant alternations after treatment no matter the dose (Figure 7B). Approximately, TTO pre-exposure caused an obviously decrease of the relative expression of TNF-α (besides 0.004%), ISG56 (0.063%, 0.031%, and 0.016%), and IL-8 (0.063% & 0.031%), compared with the infected group (Figure 7C). Meanwhile, TNF-α mRNA expression had no significant alteration after TTO treatment (0.063%, 0.031% & 0.016%), and the expression of IL-8 mRNA was similar (0.063%) (Figure 7C).

## Discussion

In recent years, TTO enjoys increased popularity in many topical formulation applied clinically to treat cutaneous infections and oral diseases (19, 20) and has been extensively researched in protozoan control, antibiotic alternative, and so on (21, 22). With regard to its antiviral effect, it was evidenced that 50% inhibitory concentration (IC50) of TTO for HSV was 0.0009% by plaque assay *in vitro* (23) and showed a simultaneously synergistic effect with acyclovir (24). Meanwhile, TTO was also competent to protect host cells from influenza virus subtype HIN1 infection and replication (25). To assess the anti-VSV effect of the TTO, we designed and conducted a series of experiments.

First, it was evidenced by MTT assay that the maximum safety concentration of TTO was 0.063% (wt/wt), and CC50 was 0.32%. As an obligate intracellular pathogen, the proliferation of VSV strictly depends on its internal replication in living cells (26). Therefore, the level of its replication rate is an intuition index for the study of the antiviral effect of TTO. Then the inhibitory effect of TTO to VSV was researched below the cytotoxic dose. Based on our previous observations, 100 TCID50 VSV can absolutely obtain access into Vero cells 6 h post-infection, and the replication rate would be relatively stable 13.5 h post-infection. Hence, RT-qPCR assay, WB analysis, and FCM assay were, respectively, performed 13.5 h post-infection for detecting the replication rate of the VSV.

In the present study, all results showed that TTO was notably competent to inhibit the VSV replication, on the basis of the FCM results, EC50 of TTO against VSV was 0.019%. Whatever the action mode of premedication, co-incubation, or pre-infection reveals the impressive prophylaxis, blocking, and treatment activities of TTO.

However, the magnitude of the replication rate of VSV was strictly lower than that in prophylaxis and treatment group after incubating with TTO. According to the previous reports, TTO inhibits replication of HSV-1 after a pre-incubation with TTO or during the viral adsorption, consistent to our research (23). Meanwhile, the main active compound of TTO, terpinen-4-ol, is evidenced to have the potential to combine with the membrane fusion site of hemagglutinin (27) and capable of altering the cell morphology of *Escherichia coli* and broke the integrity (28). Therefore, it could be speculated that TTO may also have the capability to reduce the activity of VSV or block the invasion of VSV, which need to be further evidenced.

In addition, TTO also exerted a remarkable inhibitory effect on the replication of VSV after infection, indicating its therapeutic potential to intracellular pathogen. It was reported that various monoterpene constituents of TTO show different degrees of killing effect on *P. falciparum*'s intraerythrocytic stage, inhibiting the endogenous development and the isoprenylation of the precursors (22), implying the underlying mechanism.

Furthermore, innate immune response plays a crucial role in the limitation of viral infection and synthesizes and secretes kinds of cytokines. In addition, these factors are not only for fighting against virus infection, but also promoting antiviral, antiproliferation, and immunomodulatory functions (29, 30). In the meantime, TTO was capable of exerting its immunomodulatory effect by inhibiting the nuclear factor κB signaling pathway (31). According to our study, the viral infection–induced up-regulation of ISG56, TNF-α, IL-8 was all down-regulated by the TTO, even recover to normal level. In addition, the regulation of inflammation indicates that anti-inflammation effect was dose-dependent.

## Conclusions

TTO exerted a significant inhibitory effect on VSV replication and the related inflammatory responses in Vero cells. Among three action modes in this study, blocking mode showed the best antiviral action of TTO in this study. This study highlights the potential use of TTO against viral infection.

## Data Availability Statement

The original contributions presented in the study are included in the article/Supplementary Files, further inquiries can be directed to the corresponding author/s.

## Author Contributions

QS conducted all experiments, analyzed the data, and wrote the draft with JH. JL conceived the idea, designed the project, and supervised the whole project. All authors commented on the manuscript.

## Funding

This work was supported financially through research projects from National Natural Science Foundation of China (32072911 and 31672595) and the Priority Academic Program Development of Jiangsu Higher Education Institutions.

## Conflict of Interest

The authors declare that the research was conducted in the absence of any commercial or financial relationships that could be construed as a potential conflict of interest.

## Publisher's Note

All claims expressed in this article are solely those of the authors and do not necessarily represent those of their affiliated organizations, or those of the publisher, the editors and the reviewers. Any product that may be evaluated in this article, or claim that may be made by its manufacturer, is not guaranteed or endorsed by the publisher.
