# Supplementary figures and images for: Intracellular Replication Inhibitory Effects of Tea Tree Oil on Vesicular Stomatitis Virus and Anti-inflammatory Activities in Vero Cells
Source: Front Vet Sci. 2021 Nov 17;8:759812. doi: 10.3389/fvets.2021.759812 (PMC8635969; doi:10.3389/fvets.2021.759812)

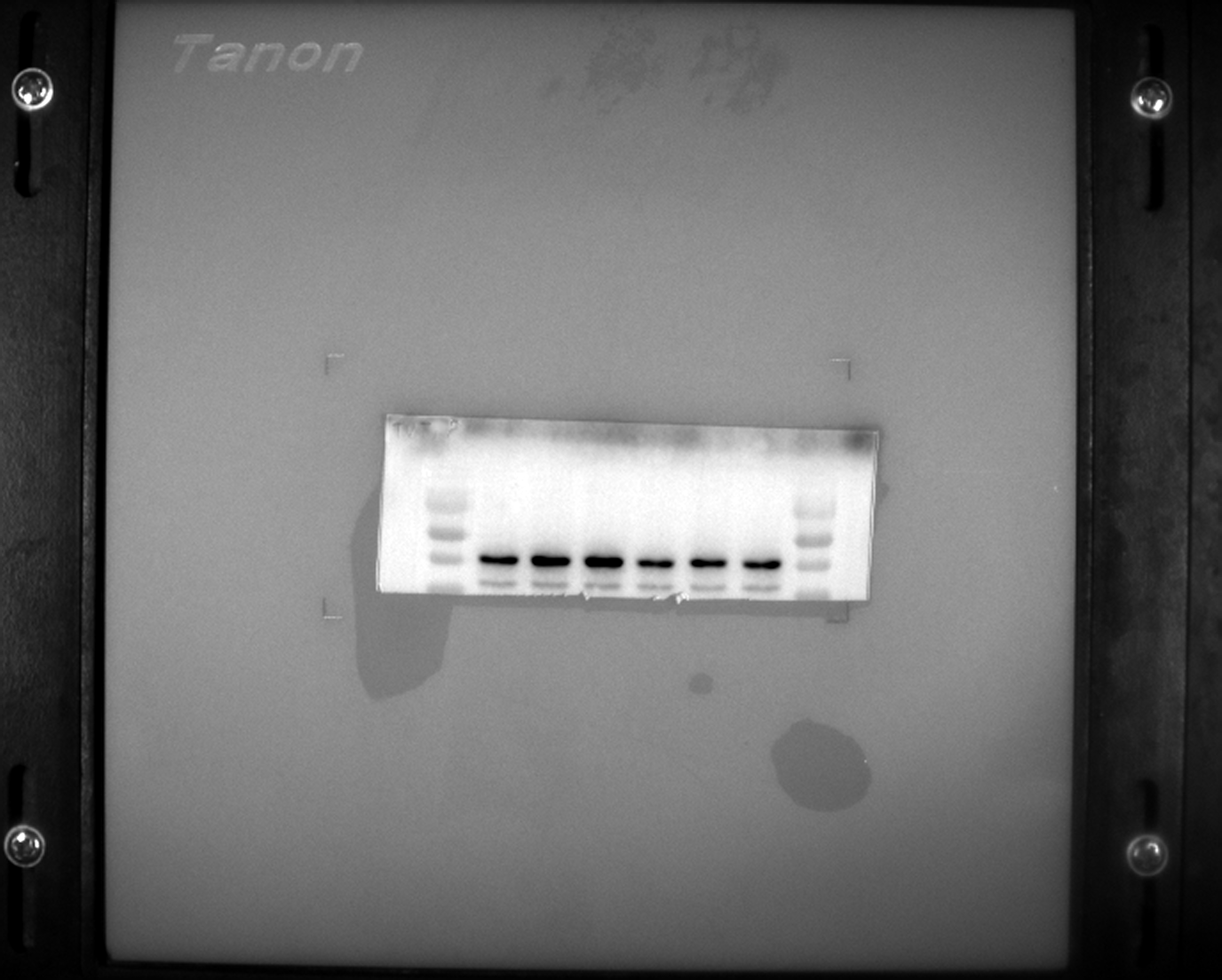

Supplement: Supplementary file 1 [file Data_Sheet_1.ZIP › RAW data/WB/20210525-TVT P-TTO-VSV-B-ACTIN.tif]

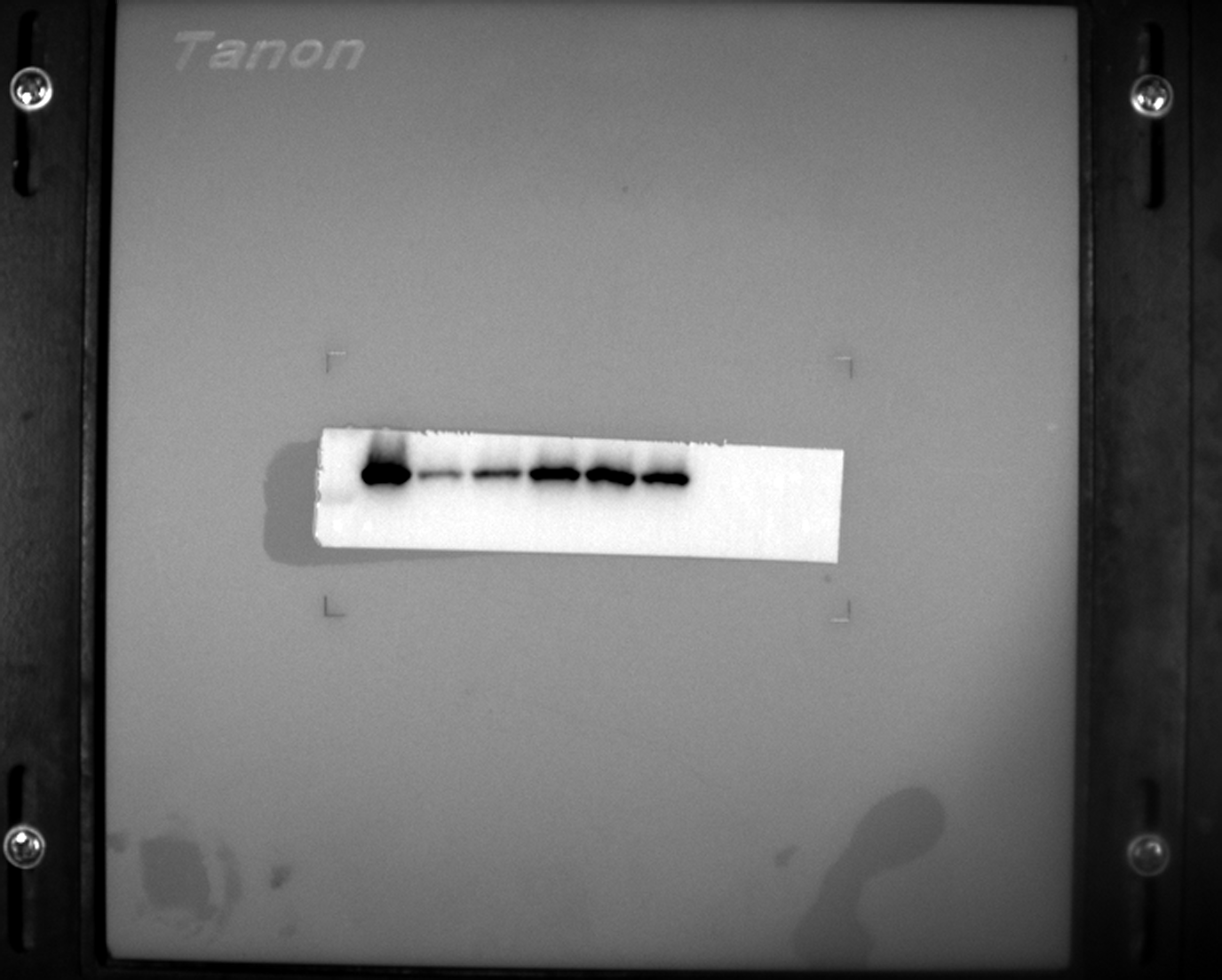

Supplement: Supplementary file 1 [file Data_Sheet_1.ZIP › RAW data/WB/gfp 3 6.23.tif]

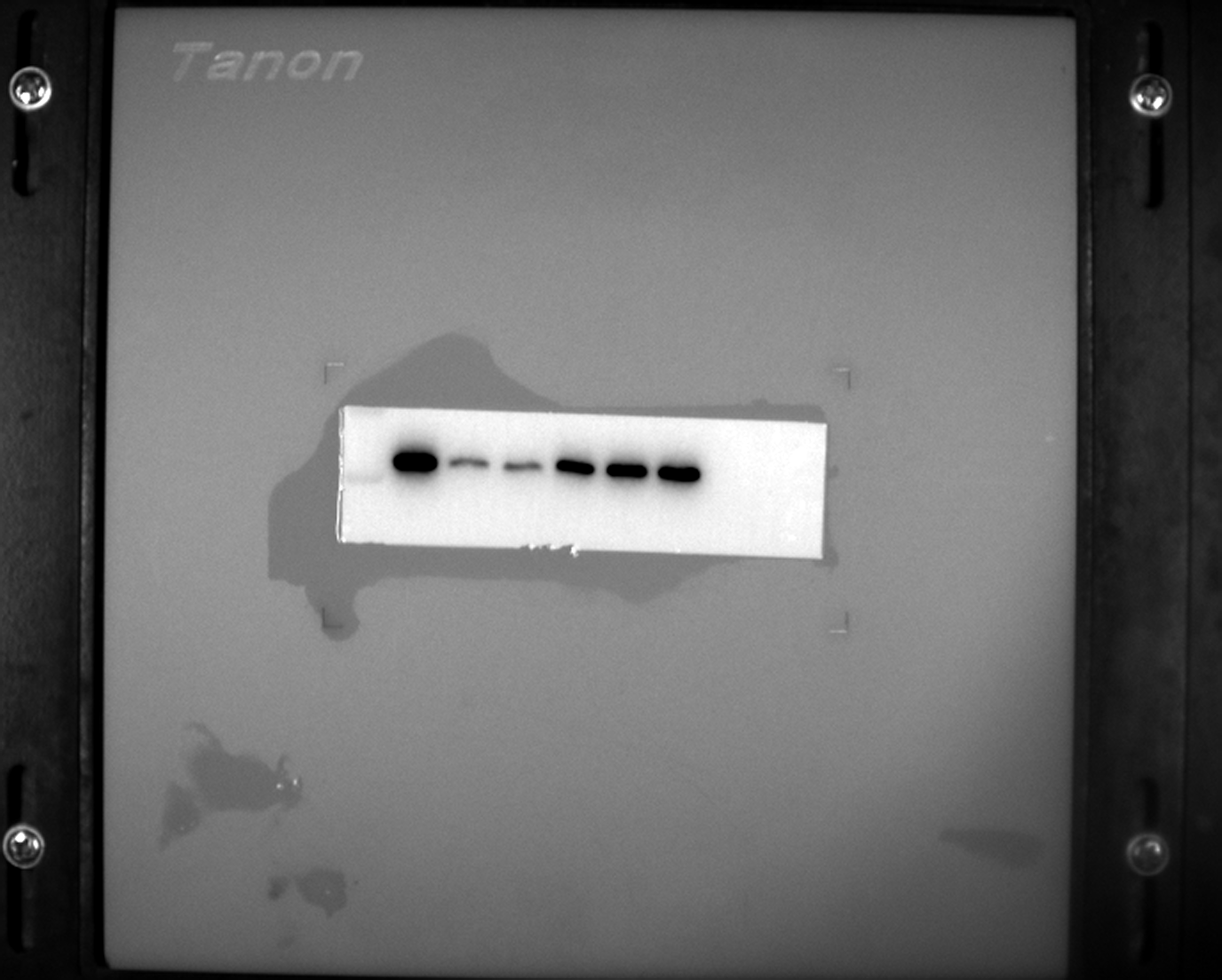

Supplement: Supplementary file 1 [file Data_Sheet_1.ZIP › RAW data/WB/gfp 6.23 12.tif]

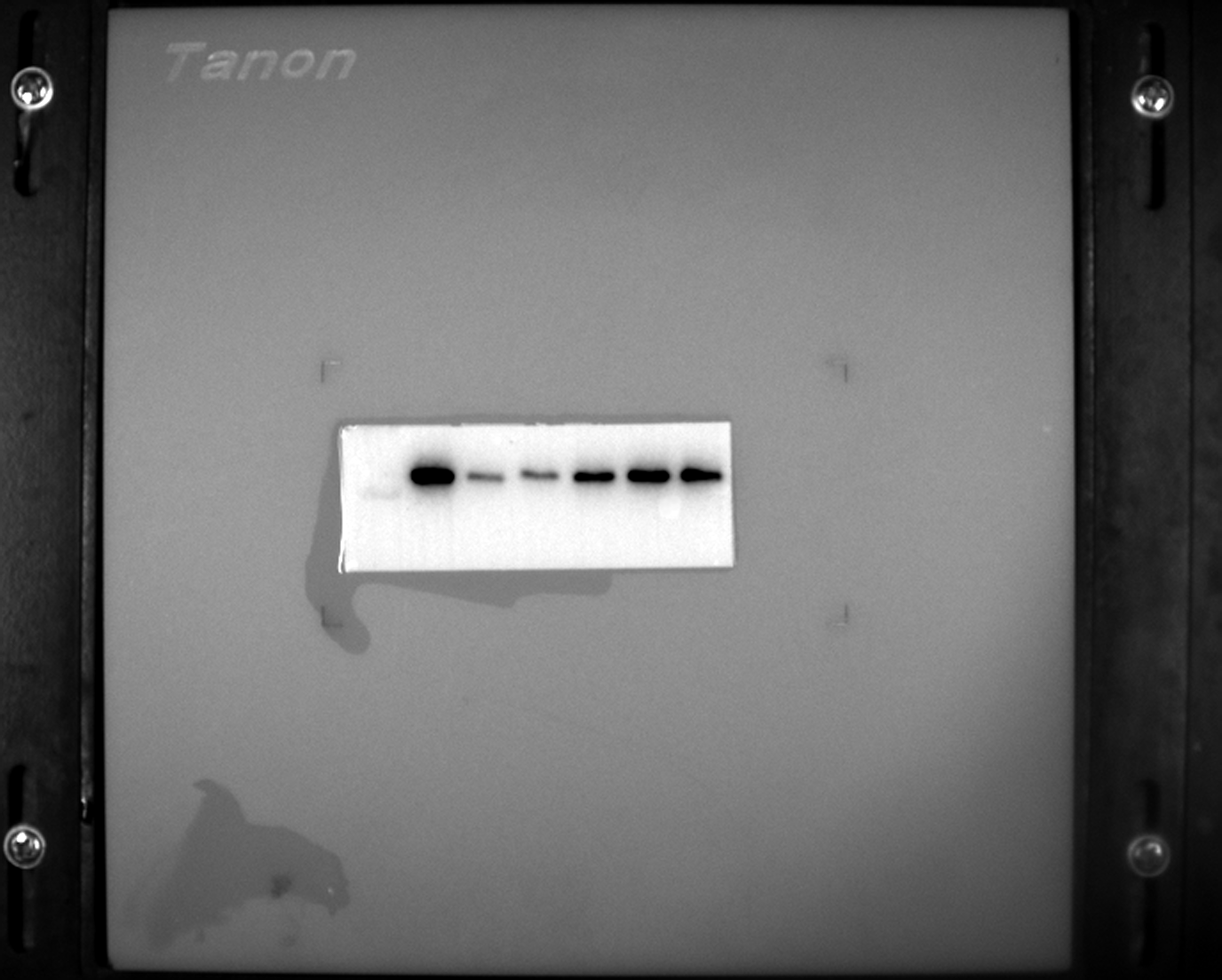

Supplement: Supplementary file 1 [file Data_Sheet_1.ZIP › RAW data/WB/gfp 6.23.tif]

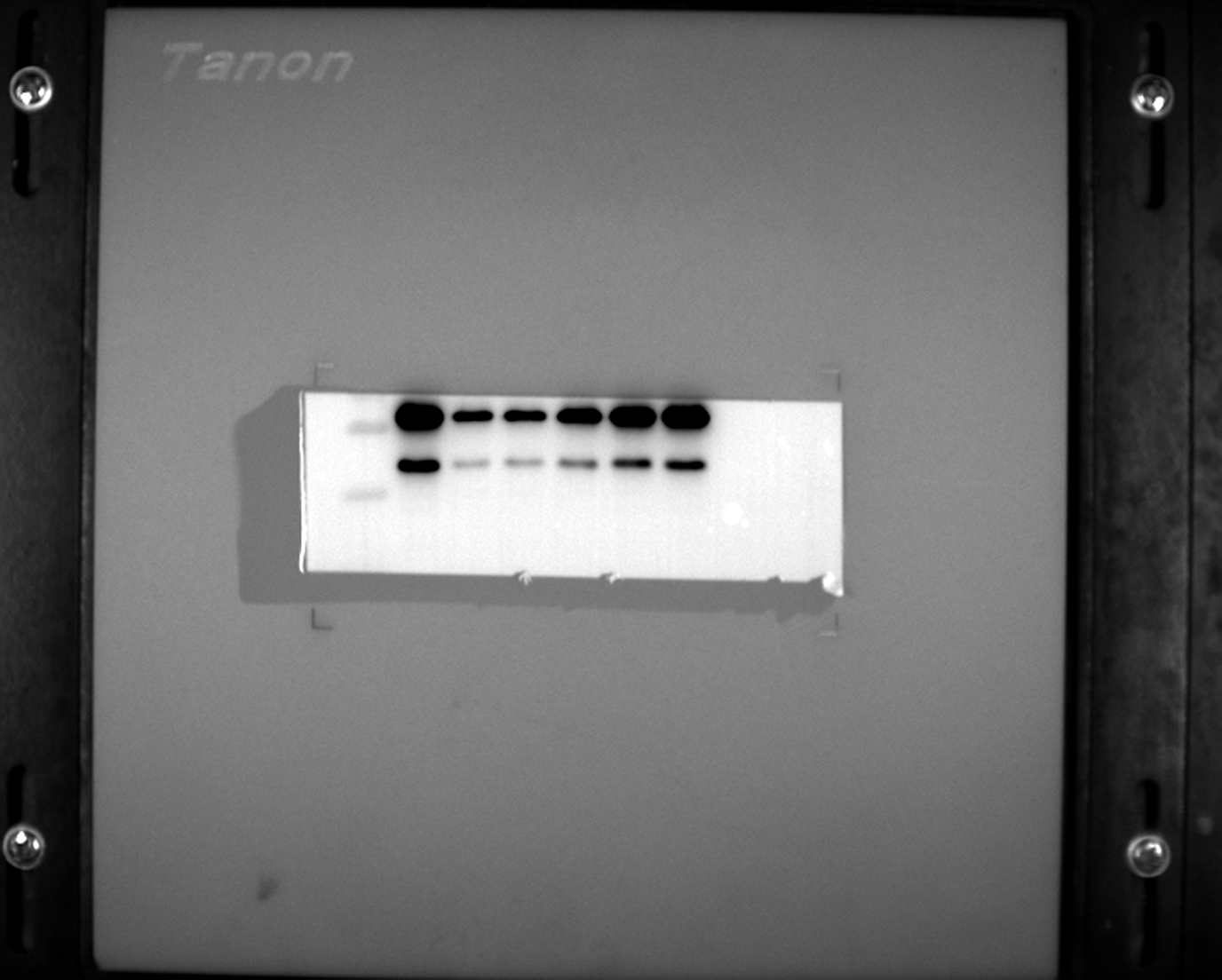

Supplement: Supplementary file 1 [file Data_Sheet_1.ZIP › RAW data/WB/PT6 6.20 GFP 2.tif]

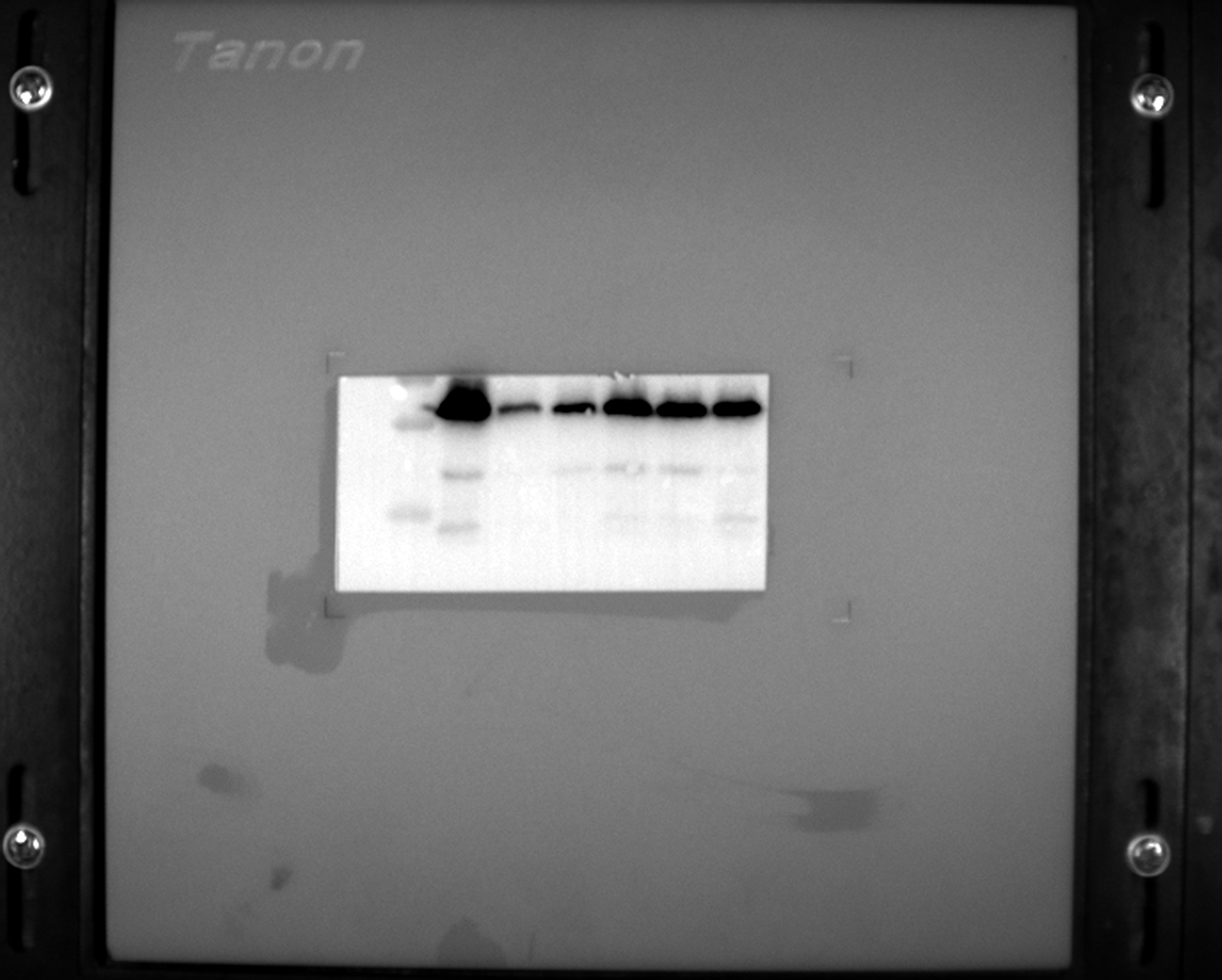

Supplement: Supplementary file 1 [file Data_Sheet_1.ZIP › RAW data/WB/PT6 6.20 GFP.tif]

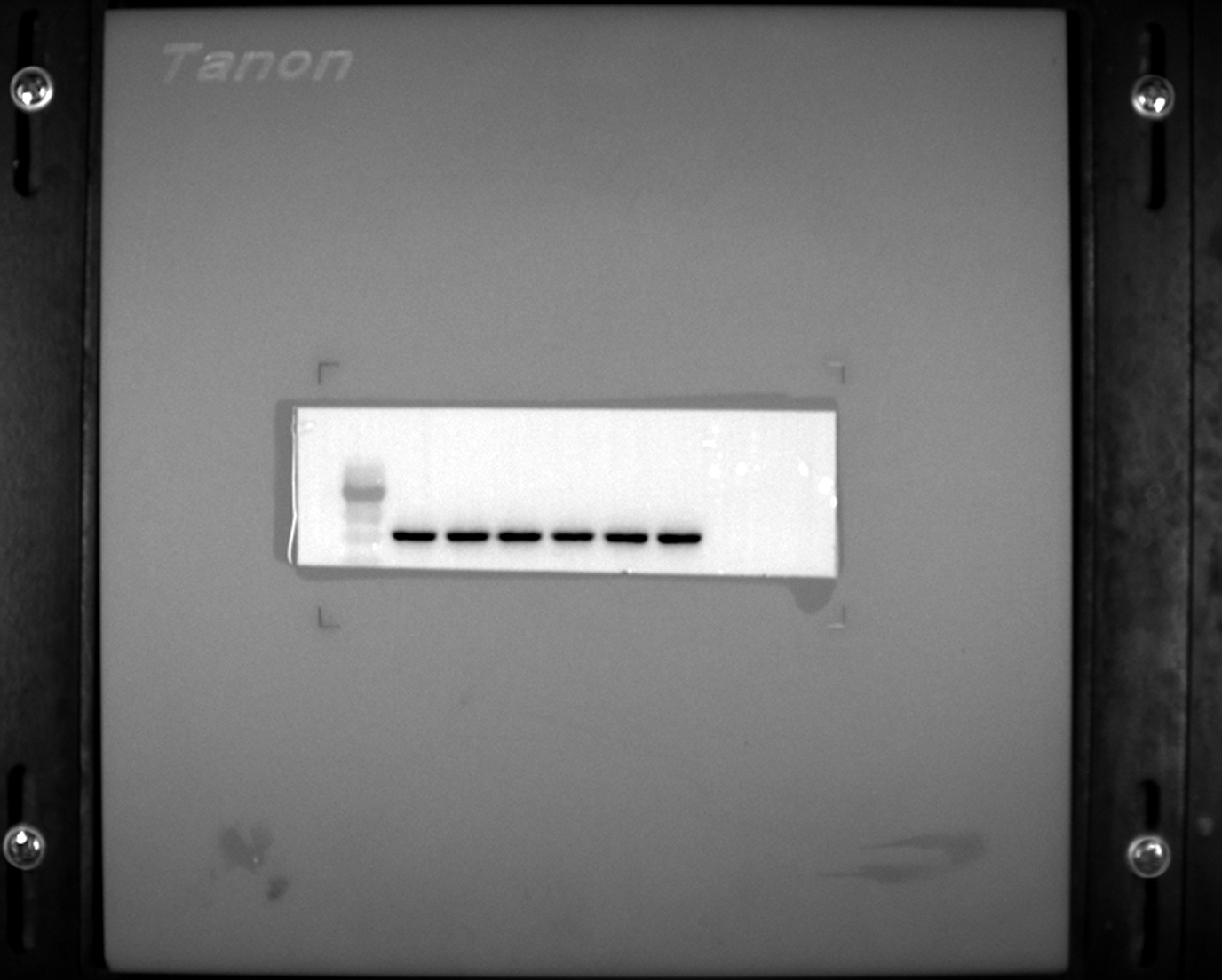

Supplement: Supplementary file 1 [file Data_Sheet_1.ZIP › RAW data/WB/PT6 B-ACTIN 6.20.tif]

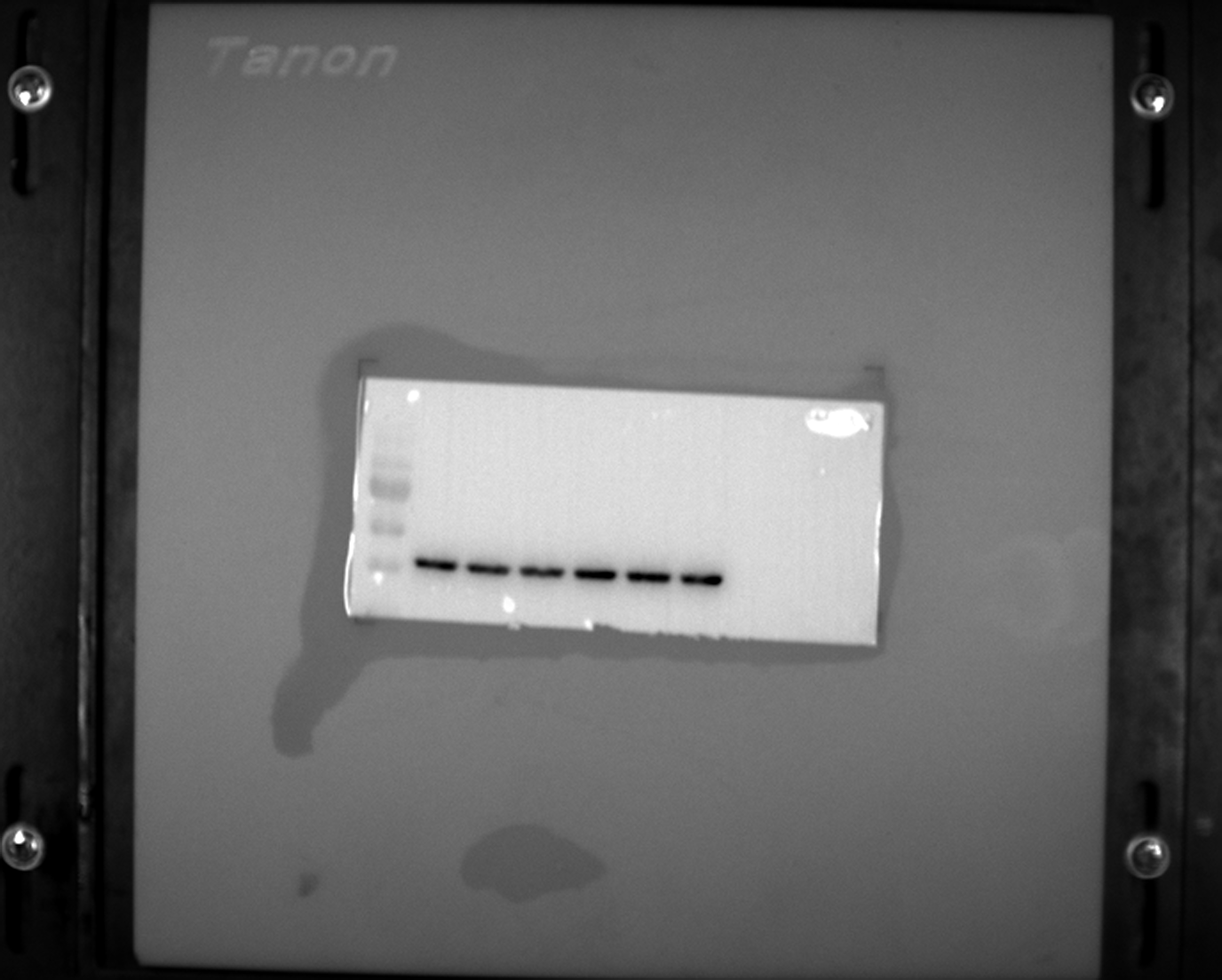

Supplement: Supplementary file 1 [file Data_Sheet_1.ZIP › RAW data/WB/pv6 1+1 b-actin 6.18.tif]

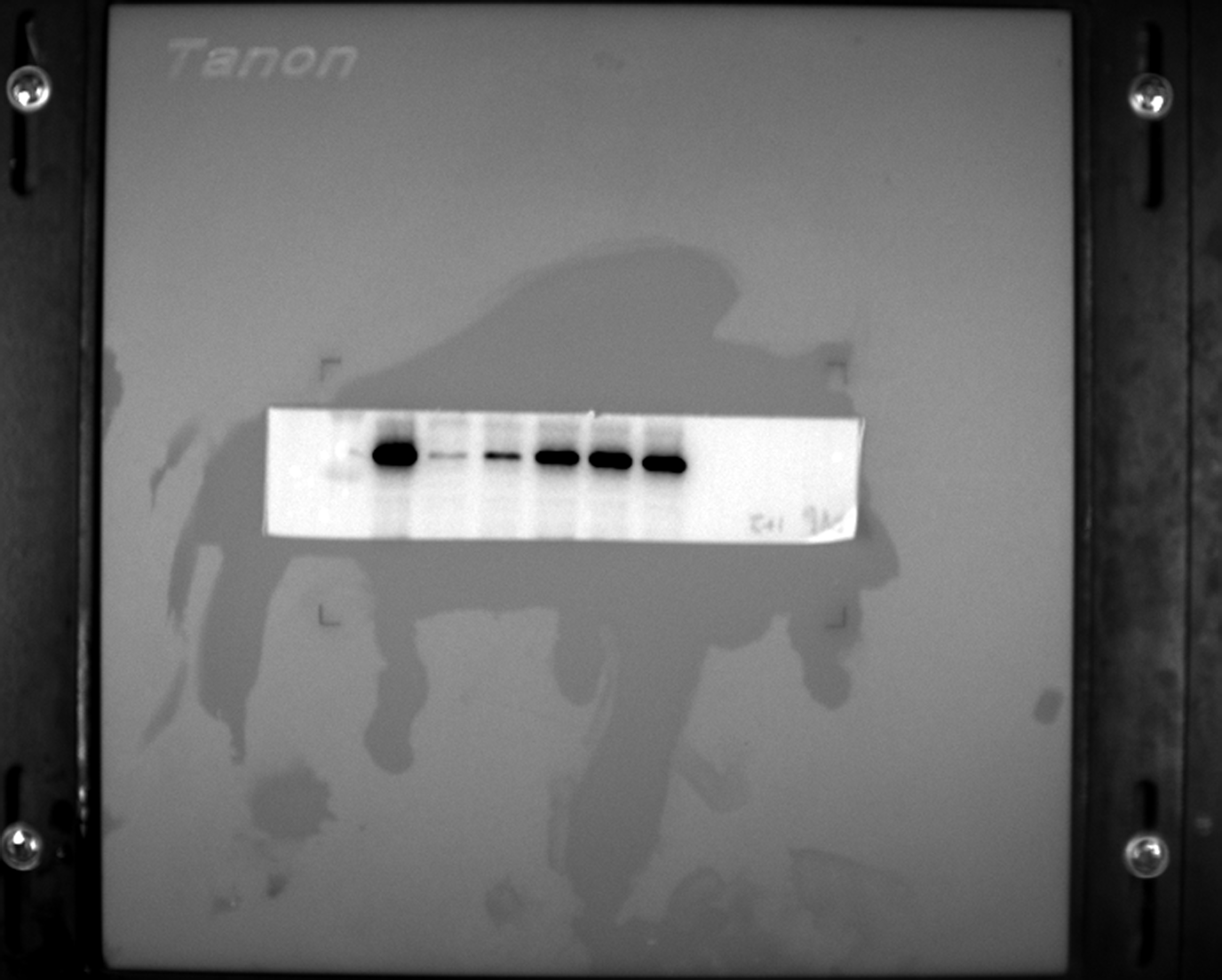

Supplement: Supplementary file 1 [file Data_Sheet_1.ZIP › RAW data/WB/PV6 1+2 6.18 GFP.tif]

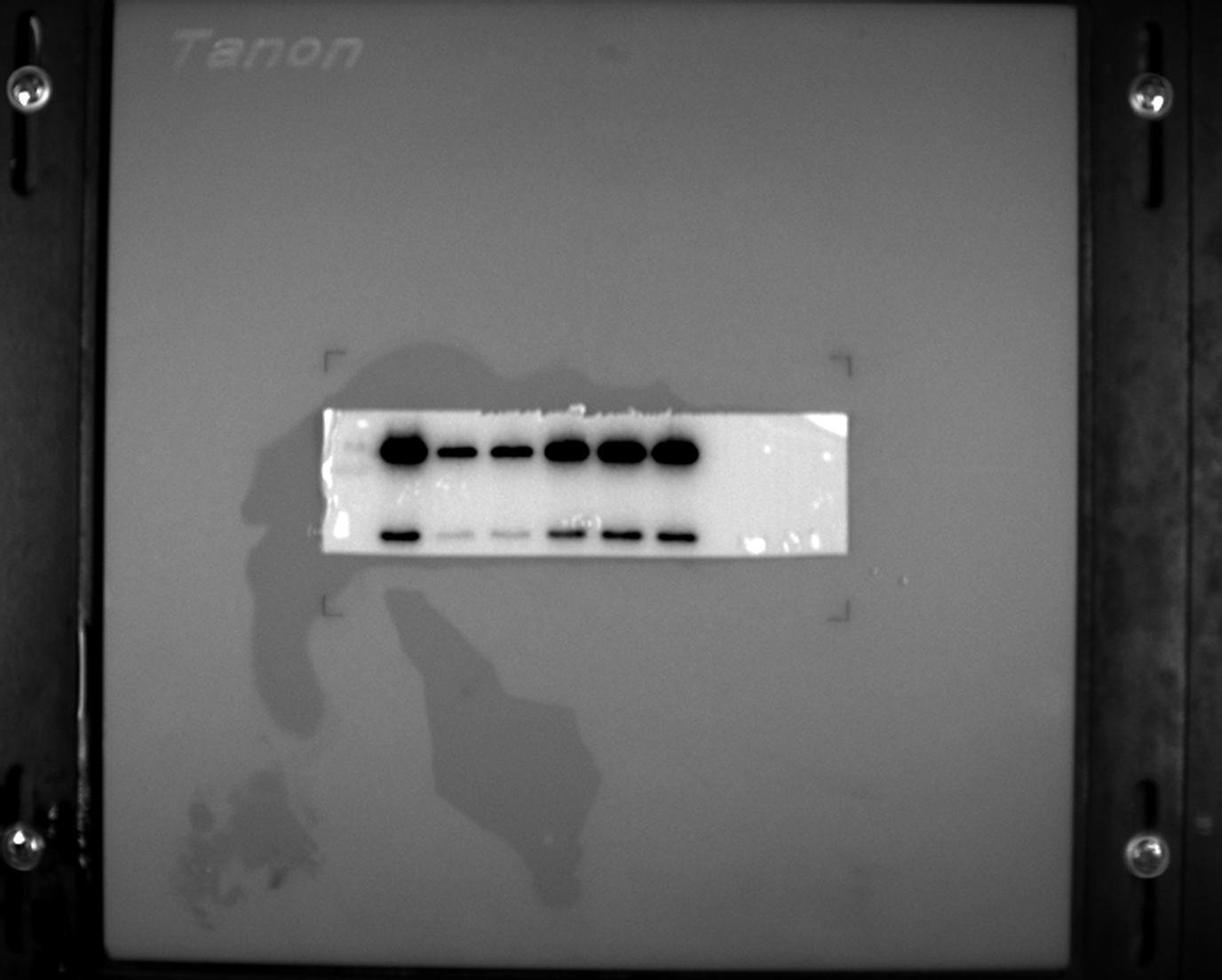

Supplement: Supplementary file 1 [file Data_Sheet_1.ZIP › RAW data/WB/PV61+1 gfp 6.18.tif]

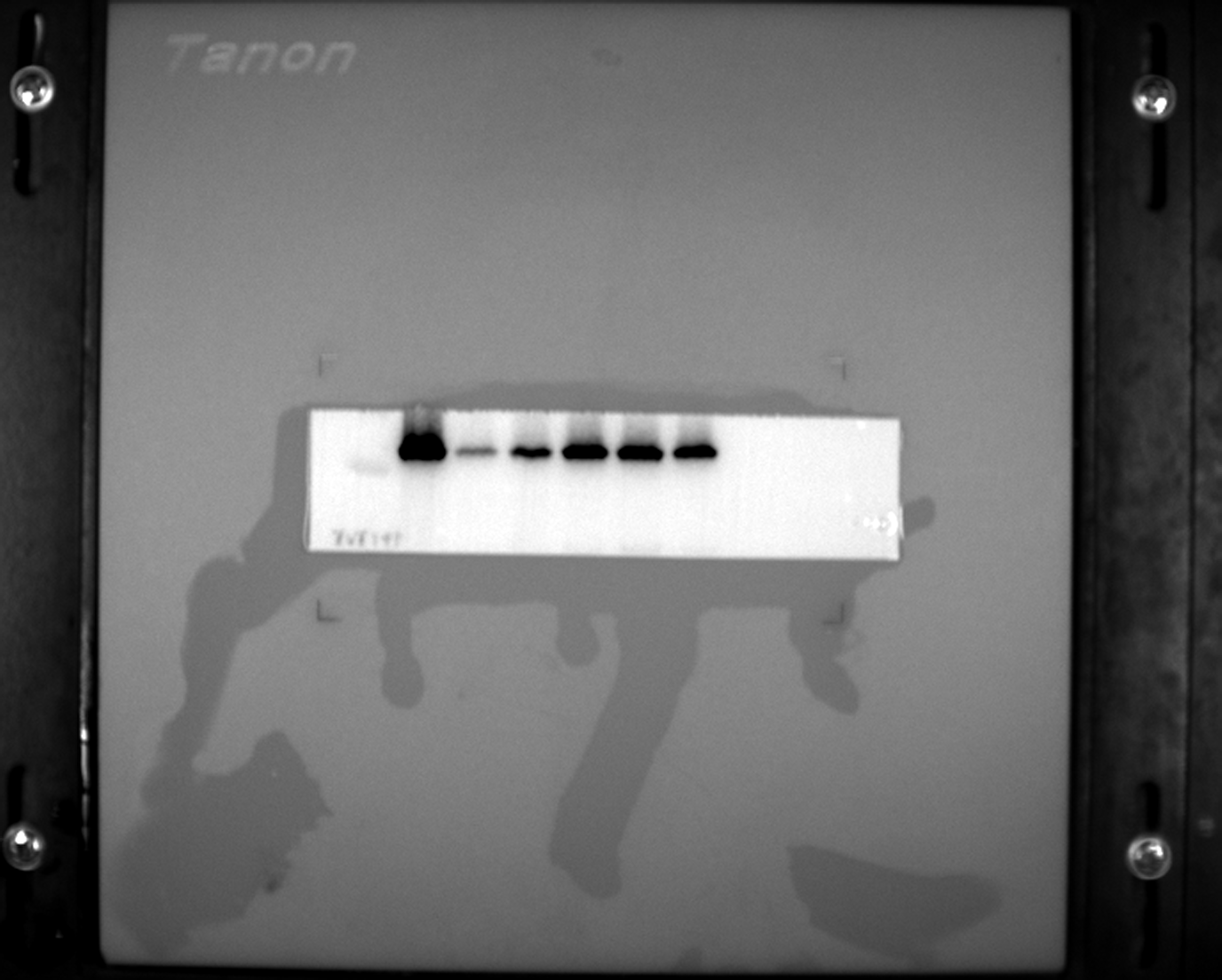

Supplement: Supplementary file 1 [file Data_Sheet_1.ZIP › RAW data/WB/tvt 1+1 6.18 gfp.tif]

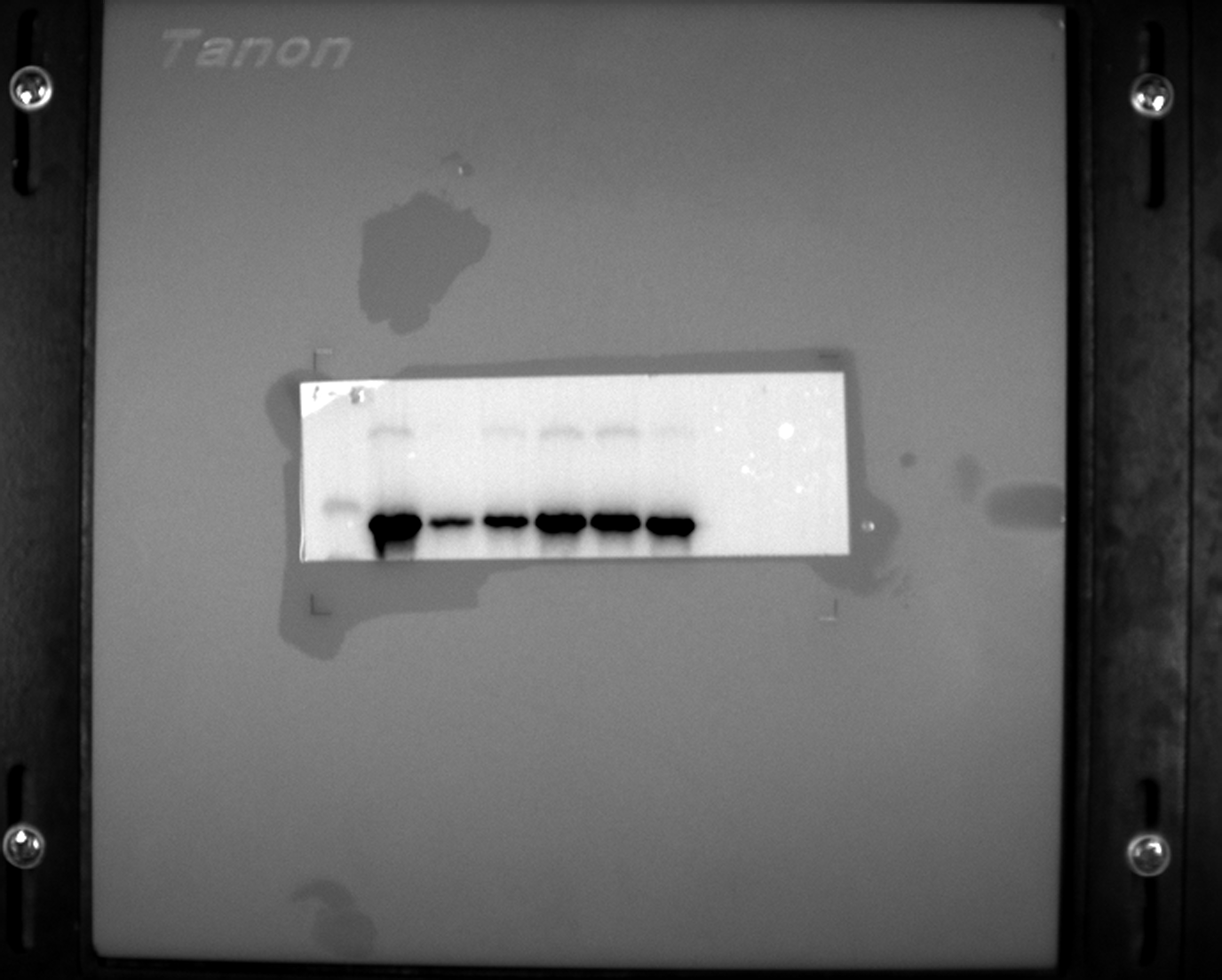

Supplement: Supplementary file 1 [file Data_Sheet_1.ZIP › RAW data/WB/TVT 1-6 GFP 6.14.tif]

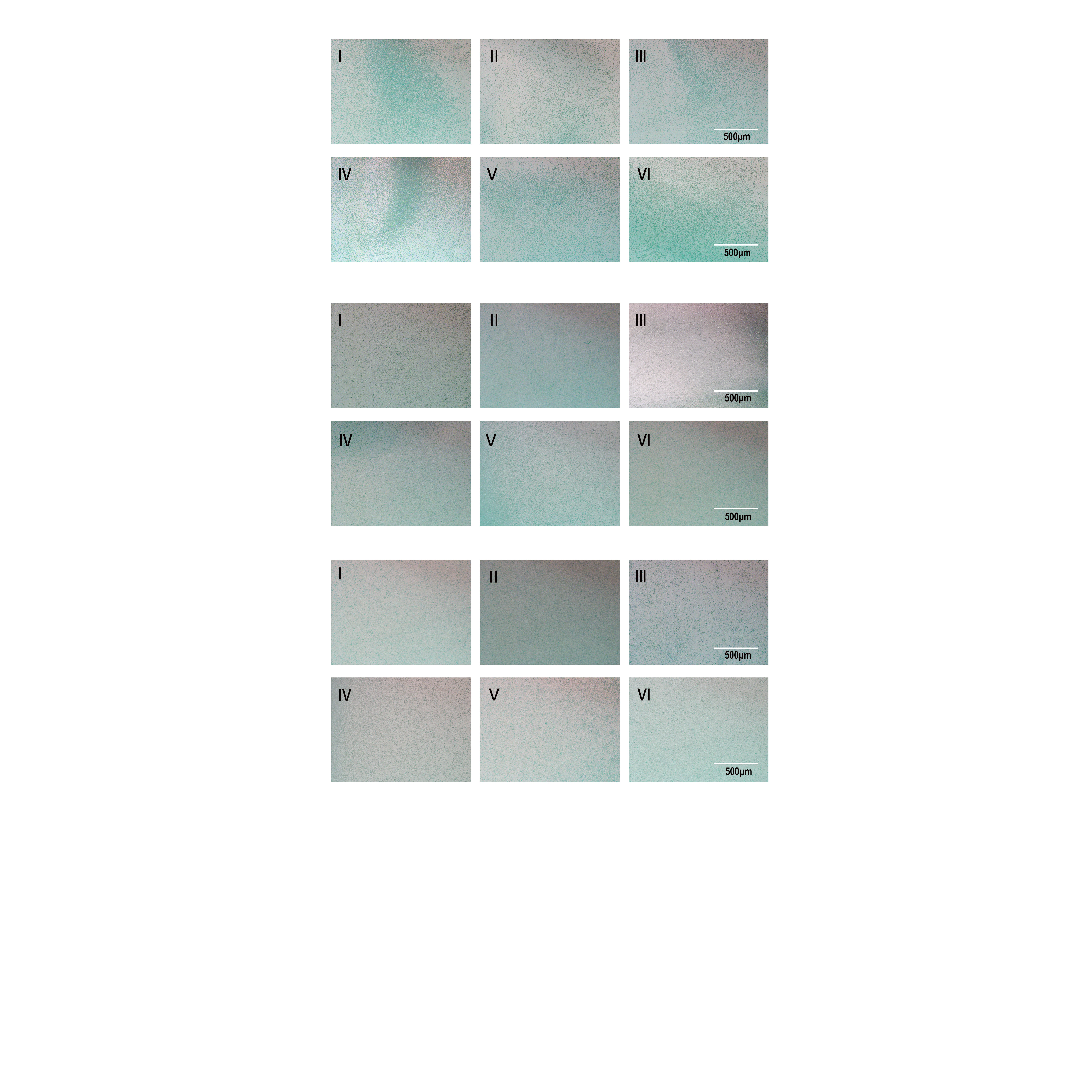

Supplement: Supplementary file 2 [file Image_1.JPEG]

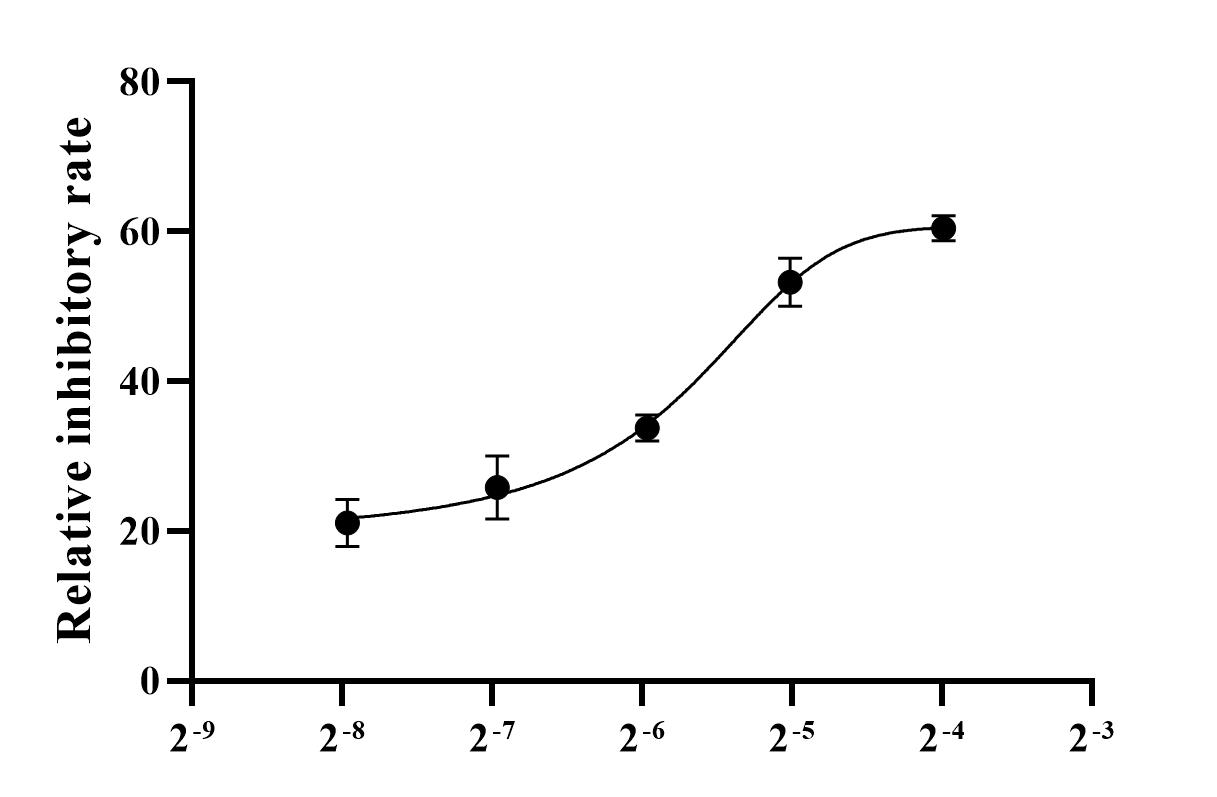

Supplement: Supplementary file 3 [file Image_2.JPEG]
